# Mitigating the Adverse Effects of Semi-Arid Climate on Capsicum Cultivation by Using the Retractable Roof Production System

**DOI:** 10.3390/plants11202794

**Published:** 2022-10-21

**Authors:** Giao N. Nguyen, Neil Lantzke

**Affiliations:** 1 Department of Primary Industries and Regional Development, 262 South River Road, Carnarvon, WA 6701, Australia; 2 Department of Primary Industries and Regional Development, 3 Baron-Hay Court, South Perth, WA 6151, Australia

**Keywords:** Gascoyne region, protected cropping, Cravo greenhouse, canopy temperature

## Abstract

Capsicum (*Capsicum annuum* L.) belongs to the *Solanaceae* family and is an economically important vegetable crop. However, the crop is very sensitive to adverse weather conditions such as high temperatures and excessive sunlight, which cause flower and young fruit to drop and sunscald to mature fruits. Using protected cultivation such as shade covers or net houses is a feasible agronomic approach to protect the crop from high light intensity, which increases plant growth, reduces fruit damage, and increases marketable fruit yield and quality. Low-cost protected cropping options such as fixed-roof net houses have proved cost-effective and suitable for fruiting vegetable production in semi-arid climatic regions. However, this structure type is unable to protect the crops from rainfall, is prone to cyclone damage and is inflexible to accommodate various vegetable crops which have different requirements for healthy and productive growth. This study was conducted in Carnarvon, which has semi-arid climatic conditions and is a key horticultural district of Western Australia, to compare the Retractable Roof Production System (RRPS) and open field (OF) conditions in the production of capsicum. The data showed that the RRPS modified the internal light, temperature and humidity in favour of the capsicum crop. The RRPS-grown capsicum had higher plant height and lower canopy temperature on hot days than those in the OF. The mean marketable fruit yield of capsicum varieties grown in the RRPS was significantly higher than those in the OF with fruit yields of 97 t ha^−1^ and 39.1 t ha^−1^, respectively, but the fruit quality remained unchanged. Overall, the data suggest that the RRPS altered the internal microenvironment and enhanced capsicum crop growth, physiology and fruit yield by setting climatic parameters to automatically control the opening and closing of the roof, the insect net and side curtains, and activation of the fogging system. The future perspective of the deployment of RRPS for capsicum production under climatic conditions in Carnarvon was also discussed.

## 1. Introduction

The global population is forecasted to reach approximately 9.6 billion people by 2050 [1]. As a result, the total food production needs to be doubled to ensure food security for the burgeoning population, which requires a steady crop yield increase at a rate of 2.4% a year [2]. However, our endeavour to increase agricultural production in general and for vegetable production, in particular, is being critically impacted by climate change [3]. In the semi-arid climatic regions, which feature high temperatures, high solar radiation in spring and summer, low annual rainfall and low relative air humidity, the weather extremes affect the growth, development and yield, as well as fruit quality of vegetables [4]. These extreme conditions will likely be exacerbated in the future by climate change. It is predicted that by 2050 the global temperature will have increased by 1.5 °C, with more frequent and intense heat waves, storm and drought events, and more unpredictable rainfall patterns, making the horticultural production in the semi-arid regions even harder [5,6].

Capsicum is an important vegetable crop due to its economic and nutritional value. According to the 2019 statistics, the total world production of capsicum was about 61 million tonnes, covering a cultivated area of about 4.5 million hectares [7]. Capsicum crops prefer mild weather conditions for healthy and productive growth and the crop is very sensitive to high temperatures and extreme sunlight. The optimum temperature for vegetative growth and fruit development of capsicum crops is proximately 20–25 °C [8]. Flower and young fruit drops are common in capsicums [9]. The cause of these phenomena is mainly due to excessive heat, lack of light, inconsistent irrigation or biological factors such as insect pests and diseases [10]. High temperature or lack of light will cause the abortion of flowers and immature fruits, resulting in yield reduction. The capsicum fruit is also sensitive to high light intensity once the fruit has reached the green ripening stage. The high light intensity plus high temperature can cause fruit burn (or sunscald) [11]. Other studies on fruiting vegetables also show that extreme sunlight can cause radiation stress on plants, critically affecting their growth and development [4]. To overcome this issue, breeders can select varieties that exhibit sufficient leaf cover that can shield the fruit from sunscald. However, these varieties tend to be prone to flower and young fruit drop [12]. Using protected cultivation such as shade covers or net houses is an agronomic approach to restrict the light intensity by 26–36%, which reduces sunscald [10,13,14].

Our previous study showed that in a semi-arid climate, excessive sunlight was the critical factor affecting the crop growth, physiology, and fruit yield of vegetables and that it could be mitigated by using fixed-roof net houses [4]. However, this structure type is unable to cover the crops from rainfall and is prone to cyclone damage. Moreover, due to its fixed roof design, it lacks the flexibility to grow various vegetable crops economically and effectively since each vegetable crop has a specific growing requirement for optimum yield and quality. The RRPS is emerging as a more advanced protected cropping option. Unlike the fixed roof net houses, RRPS can retract the roof and the side curtains to provide the plants inside with the maximum amount of solar radiation when needed. The impermeable roof can prevent unwanted rains from impacting the crop. The roof can also be closed while the side curtains are rolled up on hot days to protect plants from excessive solar radiation [15]. Using the RRPS system for fruiting vegetable production has been successfully used in many climatic regions in the world. However, the deployment of the RRPS system does not always result in better agronomic performance and a higher yield of the crop for every climate zone [16]. A thorough assessment of its usefulness in specific climate zones is required before it is recommended for commercial use. This investigation, which was an industry-oriented study and was part of the protected cropping research program for vegetable crops, coordinated by the Department of Primary Industries and Regional Development, was conducted in Carnarvon, a key horticultural district of Western Australia, where it has a typical semi-arid climate. The aim of this study was to examine if the RRPS, with a different mode of operation compared to the fixed roof net houses, can protect the capsicum crop from the climatic extremes and enhance the crop growth, and fruit yield in comparison to the field-grown control.

## 2. Results

### 2.1. RRPS Modified the Growing Environment

**Figure 1:**
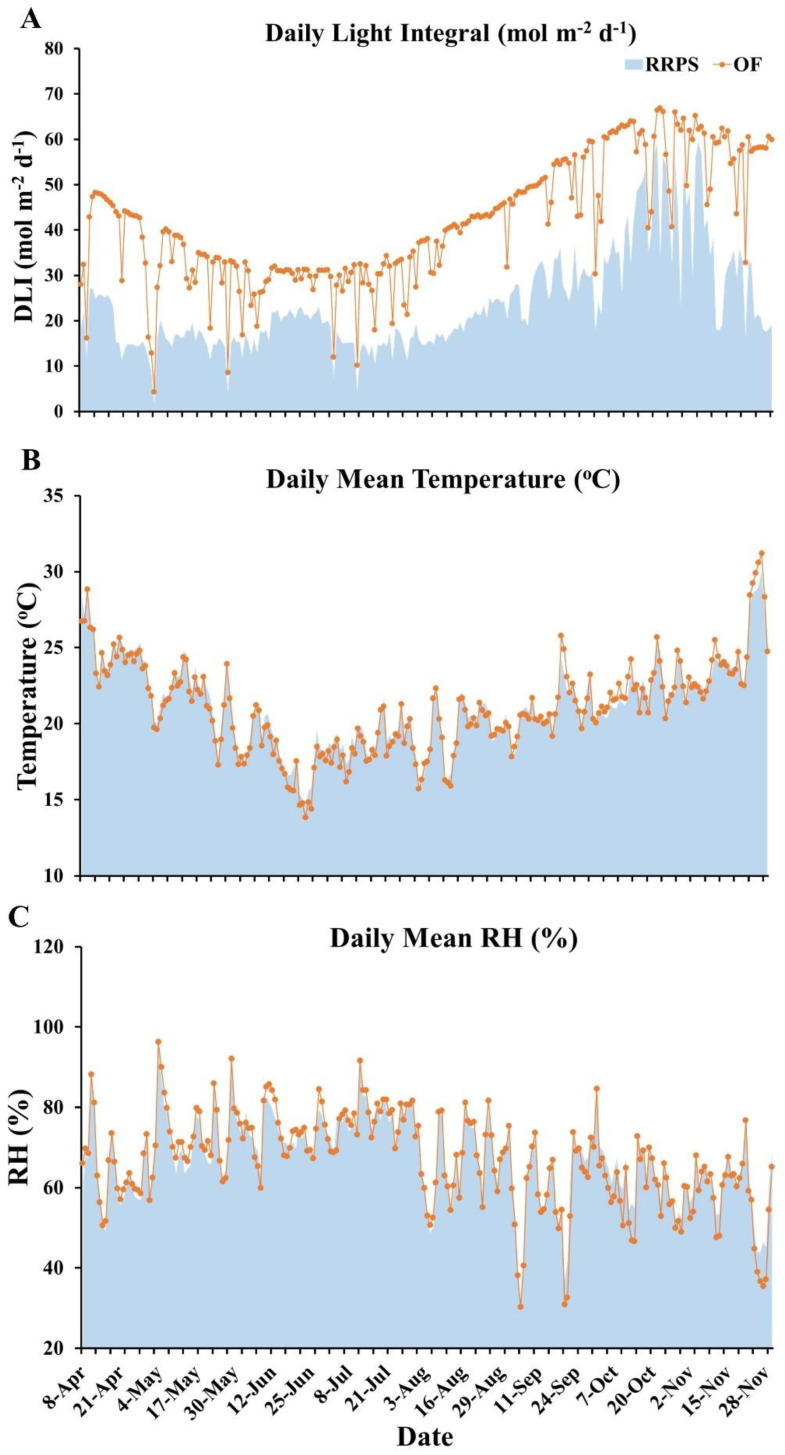
Microclimatic condition of the retractable roof production system (RRPS) and open field. (**A**) Daily light integral; (**B**) daily mean temperature; (**C**) daily mean relative humidity; OF, open field; RH, relative humidity.

In countries in the Southern Hemisphere such as Australia, the spring season starts in September and lasts until the end of November. The summer months are from December through February, followed by the autumn months, i.e., from March till the end of May. The winter season begins in June and stays till the end of August. Our experiment was planted in early April (autumn) and finished in early December (early summer) in 2021. This is the usual schedule for capsicum farming in Carnarvon. In the OF, the light intensity expressed as daily light integral (DLI) began to decrease from early autumn to the lowest level during the winter months of June and July. Then, it gradually increased from August to November (Figure 1A). The lowest average light intensity during June and July period was about 30 mol m^−2^ d^−1^. Occasionally, the light intensity dropped to very low levels on rainy days. The average light intensity in the months of October–November was extremely high, around 55 mol m^−2^ d^−1^. On several occasions, the light level surpassed 65 mol m^−2^ d^−1^, which was more than double the average light level during the winter months. The light intensity in the RRPS also varied in a similar trend to the light level in OF, but it was much lower due to the use of the roof and insect net opening and closing mechanism (Figure 1A). The average light intensity in the winter months of June–July was about 16 mol m^−2^ d^−1^. On several days between 18–29 June, the roof system was malfunctioning due to the failure of the light sensors, so the average light during this period was higher. The light intensity in the last two weeks of October was quite high with the average DLI value around 45 mol m^−2^ d^−1^. We modified the opening and closing protocol of the roof in the early days of November which resulted in a sharp decrease in the light intensity with an average value of about 30 mol m^−2^ d^−1^. Overall, the light intensity inside the RRPS was kept at a much lower level than that under the OF condition (Figure 1A).

In general, the air temperature was lowest during the months between mid-May to mid-August, which is usually the coldest time of the year in Carnarvon. The air temperature started to rise in the second half of August and peaked in late November. In contrast to the light conditions between the two environments, the average air temperature did not differ greatly between the RRPS and the OF (Figure 1B). Notably, during the period from May to August, the average air temperature in the RRPS was about one degree higher than in the OF; however, during the period starting from September to November, the temperature in the RRPS was slightly lower than that in the OF (Figure 1B).

The relative humidity (RH) was highest from May to July and decreased gradually from August to late November. Noticeably, the average RH in the RRPS was higher than in the OF due to the use of the fogging system between late September and November (Figure 1C). There were several days in October and November when the daily average RH between the RRPS and the field varied by up to 10%.

### 2.2. Chlorophyll Content

The chlorophyll content is an important indicator, showing the health status and photosynthetic capacity of the plant. The results showed that the seasonal changes affected the variability of the chlorophyll content of capsicum varieties in the two growing environments (Table 1). However, there was no interaction between the growing environment and variety in both seasons (*p* > 0.05). In the mid-season, the capsicums grown in RRPS had lower chlorophyll content as indicated by lower SPAD units (Table 1; *p* = 0.001). On average, there was approximately an 18% decrease in SPAD value for capsicums in RRPS compared to the OF. Interestingly, this trend was the opposite in the late growing season where there was about an 8.5% increase in chlorophyll content for capsicums in RRPS compared to the OF (Table 1). Among the varieties, chlorophyll content levels were similar in the mid-season. In the late season, however, Sven had a lower chlorophyll content with an average level of 61.6; while other varieties had similar chlorophyll content (Table 1).

### 2.3. Canopy Temperature

**Figure 2:**
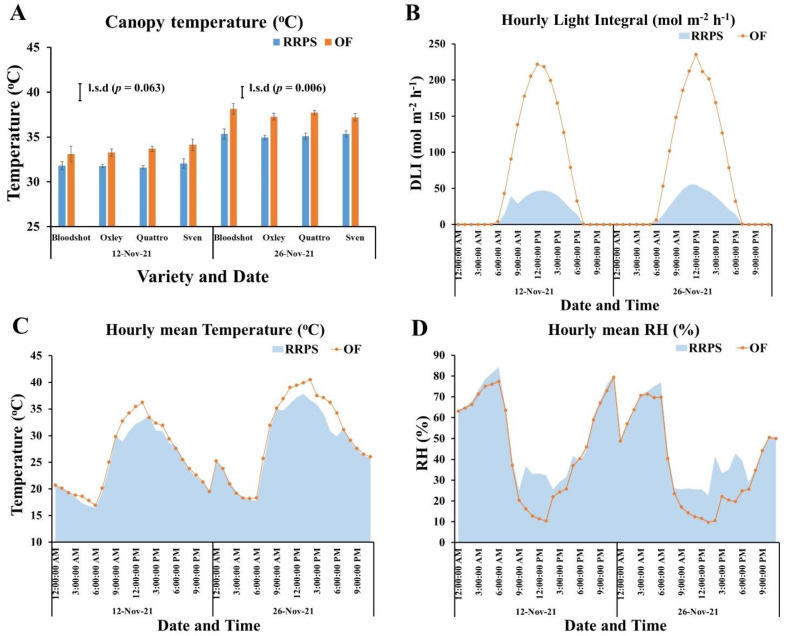
Canopy temperatures of capsicum varieties as affected by daily microclimatic conditions. (**A**) Canopy temperature; (**B**) Hourly light integral; (**C**) Hourly mean temperature; (**D**) Hourly mean relative humidity; RH, relative humidity; RRPS, retractable roof production system; OF, open field; The *p* values indicate the statistical significance at 5%; l.s.d, least significant difference at 5%.

Canopy temperature is an important indicator of how plants respond to environmental air temperatures. It also indicates how much heat stress plants are experiencing. Canopy temperature of capsicums grown in the RRPS and OF was measured on two different days (12 and 26 November 2021) when the external air temperatures were more than 35 °C (Figure 2). The results showed that the canopy temperatures of capsicum in the RRPS were approximately 1–3 °C lower than those in the OF (Figure 2A). The variation among capsicums within a growing environment was not statistically significant (*p* > 0.05). Although the canopy temperatures on 12 November 2021 were statistically marginal (Figure 2A; *p* = 0.063), the overall trend indicated that canopy temperatures in the RRPS were always lower than those in the OF. Interestingly, the hourly light integrals of the capsicum in the RRPS between 10 a.m.–2 p.m. on the two days were about fivefold lower than those in the OF (Figure 2B), which coincided with the lower hourly air temperature and higher hourly RH (Figure 2C,D). The air temperatures between 10 a.m.–2 p.m. on the two days were approximately 2–3 °C lower in RRPS than OF (Figure 2C).

### 2.4. Plant Height

Capsicum varieties grew taller, denser and had larger canopies in the RRPS than those in the OF (Table 2). However, the was no interaction between growing environments and varieties for the plant height (*p* > 0.05). The height of capsicum varieties in the RRPS were approximately 29.6% taller than those in the OF with an average height of 128.3 cm compared to 99 cm, respectively (Table 2; *p* < 0.001). On average Bloodshot was the tallest variety (122.4 cm), followed by Oxley (117.6 cm) and Sven (110.7 cm). Quattro was the shortest variety (104 cm).

### 2.5. Fruit Yield and Yield Components

There was no interaction between growing environments and varieties for the marketable fruit yield, fruit weight and rejection rate (Table 3; *p* > 0.05). However, there was an interaction between the growing environments and varieties for the fruit per plant (Table 3; *p* = 0.019).

**Figure 3:**
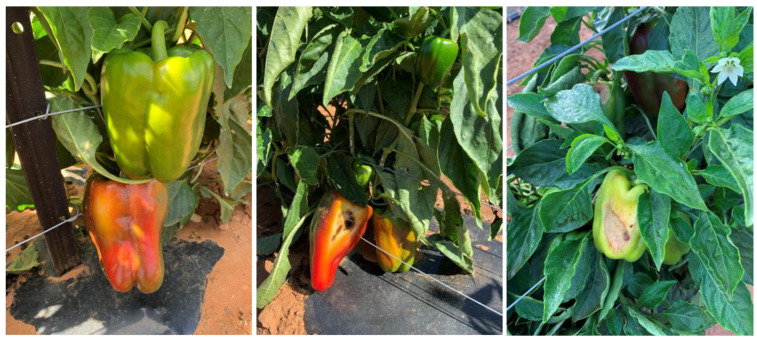
The capsicums grown in the open field: sunburnt and blemished fruit.

The average marketable fruit yield of capsicum varieties grown in the RRPS was significantly higher than those in the OF with a fruit yield were 97 t ha^−1^ and 39.1 t ha^−1^, respectively (Table 3, *p* < 0.001). Sven had the highest mean marketable fruit yield for both environments with a mean yield of 83.8 t ha^−1^, followed by Bloodshot (68.8 t ha^−1^), Oxley (62.2 t ha^−1^) and Quattro (57.4 t ha^−1^). The variation in marketable fruit yield was largely determined by the number of fruits per plant and the reject rate with the correlation coefficient (r) of 0.97 and −0.95, respectively (data table not shown). The capsicums grown in RRPS had a much higher mean number of fruits per plant (15.2 fruit plant^−1^) than those in the OF (6 fruit plant^−1^). While the average reject rates were 22.4% and 57% for capsicum in RRPS and OF, respectively (Table 3). The average fruit weights of capsicum varieties were not statistically significant between the two growing environments (Table 3). Sven had the smallest fruit weight (200.5 g); whereas other varieties had a similar fruit weight. We observed a high level of sunburnt and blemished fruit in all capsicum varieties grown in the OF (Figure 3).

### 2.6. Total Soluble Solids

There was no interaction between growing environments and varieties for the total soluble solids (°Brix) of capsicum fruits (Table 4; *p* > 0.05). Moreover, there was no significant difference in °Brix of capsicum varieties grown in the RRPS and the OF (Table 4; *p* > 0.05). Similarly, there was no significant difference in °Brix of capsicum fruits among the varieties (Table 4; *p* > 0.05).

## 3. Discussion

### 3.1. The RRPS Protected Capsicum Crop from the Adverse Weather Conditions and Improved Crop Growth and Fruit Yield

This study aimed to provide an understanding of the mechanism by which the RRPS modified the growing environment and its ability to protect a capsicum crop from adverse weather conditions in the semi-arid region of Western Australia, i.e., the Carnarvon horticultural district. In this manuscript, we have provided scientific evidence of how the RRPS altered the internal microenvironment and enhanced capsicum crop growth, physiology, and fruit yield by using a fine-tuned combination of closing and opening the roof, the insect net, the side curtains and using of the fogging system.

Using RRPS for growing fruiting vegetables commercially is not a new technology and has been applied in many regions of the world, especially in areas with extreme climatic conditions. However, before deploying this RRPS system on commercial properties, it is imperative to evaluate its suitability under local environmental conditions. Although RRPS has been successfully applied in many ecozones for various vegetable crops [17,18,19,20], several studies reported its unsuitability where the crop yield in the RRPS was not higher than those in the OF, making the return on the high investment unjustified for growers [16]. The advantage of RRPS compared to the fixed roof systems is that the roof of RRPS can be opened or closed at the users’ discretion depending on the conditions of the outside environment. If the outside environmental conditions are favourable, the roof will be opened, and the crop can take advantage of the light, humidity, and temperature of the natural environment for their growth. When the outside environment is not favourable, the movable roof and side curtains can be partially or completely closed, helping crops avoid adverse weather conditions such as heavy rain, hail, hot temperature, and extreme sunlight.

In this study, our RRPS system modified the microenvironment inside the greenhouse. In particular, the RRPS system reduced the amount of radiation entering the greenhouse significantly (Figure 1). The average night time temperature was slightly warmer than the OF (Figure 1). Increasing night time temperature in winter and decreasing daytime radiation (especially in spring) helped the capsicum crop grow better as indicated by the higher chlorophyll content, the increased plant height and the greater fruit yield (Table 1, Table 2 and Table 3). In addition to the advantages mentioned above, the RRPS system did not affect the nutritional quality of the fruits (Table 4), which has been previously observed when crops were grown in conventionally fully controlled glasshouses [21].

Another advanced feature of the RRPS system is that under the low humidity, high temperature and high radiation conditions of semi-arid climates, it can increase air humidity, limit the amount of radiation on the plant and reduce the canopy temperature (Figure 2). As a result, the level of radiation stress was reduced, plants were growing taller (Table 2), and fruits were not sunscalded (Figure 3). In the current study, the average reject rates were 22.4% and 57% for capsicum in RRPS and OF, respectively. This result was supported by a previous study conducted by Day [11], where the field-grown capsicum had 52% rejected fruits compared with 23% in the shade.

### 3.2. The Combining Effects of Light, Temperature and Humidity towards the Growth, Physiology, Fruit Yield and the Role of RRPS

As discussed above, the fruit yield of capsicum is determined by the plant density, the number of fruits per plant, the fruit weight and the rejection rate. To increase the fruit yield, it is necessary to improve these productivity components in a reasonable proportion. The influence of environmental factors such as light, temperature and humidity greatly impact these productivity components and the effect of these factors on the growth, development and yield of capsicum cannot be considered in isolation but must be assessed as a combined relationship.

The plant growth rate, the number of shoots, the number of fruit and the yield of capsicum are closely related to planting density both in the field and especially in protected cropping including RRPS [22]. Therefore, maintaining an optimal planting density is essential to take advantage of the available area of protected cropping and ensure optimum yield and fruit quality [23,24]. Planting density should be compatible with the amount of light, temperature and humidity provided by the protected cropping system and the care, pruning and pest control techniques [25,26]. The fruit number per plant is greatly affected by the plant’s ability to set flowers and develop fruits. In semi-arid conditions, the fruit set is greatly influenced by light and temperature factors. Excessive light causes the plant to suffer from radiation stress. However, under low light conditions, the plant will produce more stems and leaves, and the amount of fruit will decrease [27]. Under shading conditions, the plants will have an increased amount of ethylene, and this leads to an increased rate of flower and fruit drops. Vegetative growth and yield of capsicum are strongly correlated with mean day temperature, the effect of the temperature difference between day and night does not significantly affect vegetative growth and yield [28]. Daytime temperatures that are too high (> 34 °C) for extended periods increase the rate of flower and fruit drops [29,30]. Temperatures below 10 °C increase the male sterility rate and the number of pollinated flowers decreases, reducing the number of fruits formed [7]. Air humidity in the range of 50–80% does not affect the vegetative growth of capsicum. However, average fruit weight is affected by moisture at night and fruit set by humidity during the day [28]. Fruit sets may increase significantly if the air humidity is high during the day and the number of fruits per plant will increase if the humidity is not low at night [31].

Overall, in semi-arid climates such as Carnarvon, the RRPS system is technically well suited to growing fruiting vegetables. The RRPS system can modify light, humidity, and temperature conditions (Figure 1 and Figure 2), which can promote the growth, development, and yield of capsicum and other fruiting vegetable crops (Table 1, Table 2, Table 3 and Table 4).

### 3.3. Deployment of RRPS in Conjunction with the Use of Suitable Genetic Materials and the Best Agronomic Practices for Yield Improvement under the Semi-Arid Conditions

The key feature of RRPS is its adaptability to various crops’ growth requirements in different growing seasons as well as its ability to withstand severe storms or cyclones. Due to global warming and climate change, semi-arid climatic regions such as Carnarvon might experience more extreme weather conditions such as droughts, heat waves, strong winds and cyclones. Simple protected cropping structures such as net houses might not be able to shelter the crops from such adverse weather conditions and crops are likely to be destroyed if cyclones occur.

RRPS can potentially alter the existing cropping system of a horticultural production area. It can extend the growing season of vegetable crops in the Carnarvon region making fresh produce more competitive in the market, targeting higher-priced market windows. The weather conditions in Carnarvon during February are often very hot, with low humidity, excessive sunlight, and strong wind. Using the RRPS can allow the planting of capsicum early in February, enabling early harvests at the beginning of May for sales in the Perth market, bringing greater profit to growers. Without a protected cropping structure that can increase humidity and reduce the heat and light levels like an RRPS described above, the capsicum crop is unlikely to grow healthily and productively.

The RRPS is a protective system which can provide protection to a crop under certain weather conditions. However, to maximise the potential of the structure it is necessary to apply a comprehensive approach such as using heat-tolerant capsicum varieties which are capable of flowering and fruiting well under hot weather conditions. Other agronomic approaches such as the application of growth regulators that help reduce flower and young fruit drop under high temperatures can be used in conjunction with the RRPS [27,32].

Using the RRPS system also allows growers some flexibility to grow in soil or soilless media. This important feature makes RRPS advantageous over the conventional net houses in semi-arid regions, which were described previously by Nguyen et al. [4], where soilless growing media cannot be used due to water-permeable roofing. In locations where there are serious problems of soil-borne pathogens, the ability to apply soilless cultivation is essential. A fully circulated closed hydroponic system can protect plants from soil-borne pathogens and has the ability to recycle wastewater, increasing water and fertiliser use efficiency.

There are many different types of protected cropping structures, with the level of sophistication generally increasing the cost. One of the biggest disadvantages of the adoption of the RRPS (such as the Cravo greenhouse) is the high initial investment cost with an estimated value of AUD 1 million per one-hectare greenhouse (Miller per. comm.). Therefore it is a prerequisite for commercial growers to conduct an economic analysis before they decide to invest in a protected cropping option [33]. Additionally, the operation of the RRPS is technically more sophisticated than a conventional net house, with higher maintenance costs. Thus, identifying the most suitable and cost-effective protected cropping system for vegetable production in semi-arid climates is still an open-ended question and will be considered in future investigations.

## 4. Materials and Methods

### 4.1. Experimental Conditions

**Figure 4:**
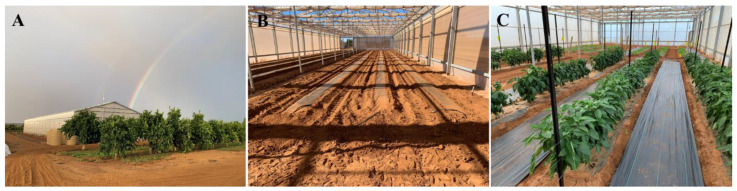
Capsicum experiment in the Retractable Roof Production System (RRPS). (**A**) External view of the RRPS; (**B**) internal view of the RRPS and the raised beds covered with plastic mulch; (**C**) the capsicum crop with horizontal trellising system.

The trial was conducted at two growing environments, i.e., the RRPS (Figure 4A,B) and the OF at the Carnarvon Research Station, Department of Primary Industries and Regional Development (24°51′18.00″ S latitude, 113°43′46.33″ E longitude) in 2021. The experiment started on 6 April 2021 and finished on 8 December 2021. The trial site was located on red silty loam soil. The RRPS is a Cravo “Rafter” Retractable Roof Cooling House, designed and constructed by Cravo Equipment Ltd. (Brantford, ON, Canada). The RRPS is 14.6 m wide and 54.9 m long and 4.3 m high and was designed to endure the wind load of up to 177 km/h wind when the roof is fully closed.

The experiment was designed similarly to our previous experiment [4]. In brief, the experimental design was a Randomised Complete Block Design (RCBD) with three replicated blocks in each growing environment, the varieties are randomised within the replicated blocks. Before planting, raised beds of 60 cm wide and 10 cm high were prepared, spaced 1.2 m apart. Polyethylene mulch and drip irrigation tape were laid at bed forming (Figure 4B). The dimension of each experimental plot was 60 cm wide × 4.2 m long. One-month-old capsicum seedlings were planted on staggered double rows on the raised beds at a 30 cm spacing between the rows and 40 cm spacing within the rows to achieve the final density of 27,778 plants per hectare. Four capsicum varieties used in this experiment were kindly provided by the seed companies, i.e., Bloodshot and Oxley of Syngenta, Sven of Rijk Zwaan and Quattro of HM Clause (Figure 4C).

The fertilizing program of the capsicum experiment is summarised in Supplementary Table S1. Monoammonium phosphate, super copper zinc molybdenum, potassium sulphate, potassium nitrate, magnesium nitrate, and calcium nitrate were applied to supply the following rates per hectare: 223 kg nitrogen, 141 kg phosphorus, 303 kg potassium, 47 kg magnesium, 169 kg calcium, 0.11 kg copper, 0.21 kg molybdenum, 0.4 kg manganese, 1.1 kg zinc. The basal fertilisation was applied before laying the plastic mulch and the weekly fertigation began two weeks after the planting. Pruning and training plants to the trellis were carried out fortnightly commencing three weeks after planting, following the standard practices in the region. Trellising keeps the plants upright and minimises lodging. In this experiment we used the horizontal trellising method, using wires to train the capsicum plants (Figure 4C). Pests and diseases were monitored weekly and controlled by using appropriate chemicals.

### 4.2. The Operation of the RRPS

The general strategy of RRPS operation was to create an optimal environment for the capsicum crop to grow healthily and productively. The opening (retracted) and closing of the RRPS’s white roof and insect net were controlled by the temperature of the black plate, which was driven by the variation of external air temperature and light levels. The retractable side curtains were also controlled by the black plate temperature. In addition, the operation of the roof and side curtains could be overridden by wind speed and rain which is monitored by an external wind and rain sensor, respectively. Subject to users’ settings, the roof and side curtains can be closed or opened if the wind speed (at a specific direction) is higher than a predefined threshold. By setting a threshold for the back plate readings and wind speeds the user can manipulate the internal air temperature, humidity, light and wind condition in the greenhouse, to some extent. The rain sensor was set so that when it rained the roof would be fully closed.

Another important concept is the setpoint which refers to the user’s defined time period within a day. We applied two setpoints during this experiment. In the first setpoint, the roof and curtains were fully opened in the early morning (7 a.m.) subject to external air temperature > 14 °C and wind conditions. In the second set point, the roof and curtains were set to close at 6 p.m. subject to external air temperature < 18 °C. This strategy ensured that the crops would receive maximum sunlight from early morning, when temperatures were rising, to retain some warm air trapped inside by the roof and curtains closing in the late afternoon. During the day, if the black plate temperature was 32 °C, the roof would be opened 40% (or closed 60%). However, if the internal humidity became less than 40%, the roof would be opened by 10%. At this point, all curtains will be fully closed, the insect net was opened 99%.

The fogger was controlled by a separate system, i.e., the Netafim irrigation controller. When the internal temperature was > 28 °C and the humidity was < 60% the fogger activated to increase the internal humidity. Fogging was manually set to be operated in sync with the operation of the roof and curtains to improve internal climatic conditions for the crop.

### 4.3. Weather Conditions

A micro weather station Watchdog Model 1450 of Spectrum Technologies, Inc. (distributed by John Morris Group, Sydney, Australia) was installed inside the RRPS. A second identical micro weather station was installed outside the RRPS. We reproduced some data collected from this weather station with permission from Nguyen et al. [4]. The weather station includes built-in temperature and relative humidity sensors. An external quantum light sensor to measure photosynthetically active radiation (PAR) was also installed. All measurements were automatically logged every 15 min. These records were downloaded monthly and used to calculate DLI, daily mean temperature and daily mean RH as described previously by Nguyen et al. [4]. Hourly mean light integral, hourly mean temperature and hourly mean relative humidity were calculated based on the average readings of hourly records.

### 4.4. Plant Height Measurement

The height of capsicum plant was determined just before the experiment completion. Five plants per experimental plot were randomly selected for the measurement. The height was measured from the ground to the topmost terminus of the plant as described previously by Nguyen et al. [4].

### 4.5. Leaf Chlorophyll Content

Chlorophyll levels were measured by a portable SPAD meter (SPAD-502 Plus; Spectrum Technologies Inc., Aurora, IL, USA) following the protocol described in Nguyen et al. [34]. The second youngest mature leaf of the six representative capsicum plants per experimental plot was selected for the measurement. We determined the chlorophyll content in the mid-season (early August 2021) and late season (early November 2021).

### 4.6. Canopy Temperature

Canopy temperatures of capsicum were measured several times during November 2021 by using a handheld IRT 6210L, model Agri-Therm III (Everest Interscience Inc., Tucson, AZ, USA). The measurements were taken on a hot, sunny day between 12 p.m.–3 p.m. The IRT was aimed at the canopy at a 30° angle from a 0.5 m distance and held for about six seconds to achieve a stable reading. Six readings were randomly taken per experimental plot.

### 4.7. Marketable Fruit Number and Yield

**Figure 5:**
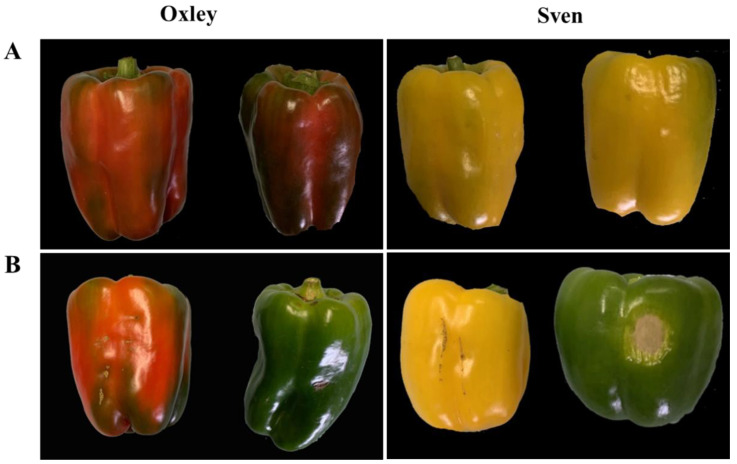
Classification of capsicum fruits. (**A**) marketable grade fruits; (**B**) reject fruits.

Mature fruits of six representative plants per experimental plot were harvested at 10–14-day intervals. Fruits of reasonably good size, similar to the market standards and free from marks, blemishes and insect bites were graded as marketable; the remaining were classified as rejects (Figure 5). The number, the weight of marketable fruit, the average fruit weight and the reject rate from multiple harvests were determined. The total marketable fruit weights were used to calculate the fruit yield per ha.

### 4.8. Total Soluble Solids

The juice was extracted from five representative capsicum fruits per plot by a juice extractor. The total soluble solids (°Brix) were measured from fruit juice using a digital refractometer Atago RP-1 (Atago Co., Ltd., Kyoto, Japan) as described previously by Nguyen et al. [4].

### 4.9. Statistical Analyses

Two-way analysis of variance (ANOVA) was performed to analyse environmental effects, varietal effects and their interaction, using GENSTAT statistical software version 21 (VSN International Ltd., Hemel Hempstead, UK). The blocking structure was specified in such a way that the environmental effect was assessed based on the replicate means within the environmental blocks [4]. Means were compared at 5% probability according to least significant difference (l.s.d) test.

## 5. Conclusions

In this research, we have provided experimental evidence demonstrating the outperformance of the capsicum varieties in the RRPS against the OF control under the semi-arid climatic conditions in Carnarvon. The RRPS could alter the internal growing environment in favour of the capsicum crop, i.e., increasing the humidity, and reducing excessive coming light and temperature, resulting in better crop growth and higher marketable fruit yield. These findings will assist capsicum growers to decide if the RRPS technology is suitable for their commercial production and investment capability. With better illustrations of the return on investment, this technology might be the future choice of capsicum growers in semi-arid regions in the years to come. It is recommended that further studies are conducted to validate the application of the RRPS to other fruiting vegetables.

## Figures and Tables

**Table 1:** Chlorophyll content of capsicum leaves is indirectly represented by SPAD units at the mid- and late-growing seasons.

Variety	Mid-Season			Late-Season		
	OF	RRPS	*Mean*	OF	RRPS	*Mean*
Bloodshot	71.1	58.4	64.8 ^a^	63.3	66.8	65.1 ^b^
Oxley	71.9	57.4	64.7 ^a^	63.7	69.8	66.7 ^b^
Quattro	68.8	63.6	66.2 ^a^	63.0	66.5	64.8 ^ab^
Sven	65.4	55.6	60.5 ^a^	57.6	65.6	61.6 ^a^
*Mean*	69.3 ^b^	58.8 ^a^		61.9 ^a^	67.2 ^a^	
ANOVA	E	V	E × V	E	V	E × V
s.e.d	1.26	3.19	4.52	1.31	1.53	2.16
*p*	0.001	0.359	0.514	0.016	0.037	0.408
l.s.d (*p* = 0.05)	3.50	-	-	3.64	3.33	-

OF, open field; RRPS, retractable roof production system; E, environment; V, variety. Means of the main effects in the same row (or column) that include a common letter are not significantly different at 5%. The *p* values indicate the statistical significance at 5%; s.e.d, standard error difference of the means; l.s.d, least significant difference at 5%.

**Table 2:** The height of capsicum varieties grown under retractable roof production system (RRPS) and open field (OF).

Variety	Plant Height (cm)		
	OF	RRPS	*Mean*
Bloodshot	108.3	136.4	122.4 ^c^
Oxley	97.2	138.1	117.6 ^bc^
Quattro	92.2	115.8	104.0 ^a^
Sven	98.3	123.1	110.7 ^ab^
*Mean*	99.0 ^a^	128.3 ^b^	
ANOVA	E	V	E × V
s.e.d	1.7	4.7	6.6
*p*	<0.001	0.011	0.284
l.s.d (*p* = 0.05)	4.8	10.2	-

Means of the main effects in the same row (or column) that include a common letter are not significantly different at 5%. E, environment; V, variety. The *p* values indicate the statistical significance at 5%; s.e.d, standard error of differences of the means; l.s.d, least significant difference.

**Table 3:** Marketable fruit yields, fruit per plant, fruit weight and reject rate of capsicum varieties grown under retractable roof production system (RRPS) and open field (OF).

Variety	Marketable Fruit Yield (t ha^−1^)			Fruit per Plant			Fruit Weight (g)			Reject Rate (%)		
	OF	RRPS	*Mean*	OF	RRPS	*Mean*	OF	RRPS	*Mean*	OF	RRPS	*Mean*
Bloodshot	41.3	96.4	68.8 ^b^	6.0	14.5	10.3 ^b^	247.5	238.2	242.8 ^b^	55.3	17.5	36.4 ^a^
Oxley	30.8	93.6	62.2 ^ab^	4.2	13.1	8.7 ^ab^	262.4	257.1	259.8 ^b^	64.0	20.9	42.4 ^ab^
Quattro	34.3	80.5	57.4 ^a^	4.7	12.2	8.5 ^a^	261.5	237.3	249.4 ^b^	58.5	30.5	44.5 ^b^
Sven	50.0	117.6	83.8 ^c^	9.0	21.1	15.1 ^c^	200.4	200.7	200.5 ^a^	50.3	20.5	35.4 ^a^
*Mean*	39.1 ^a^	97.0 ^b^		6.0 ^a^	15.2 ^b^		243.0 ^a^	233.3 ^a^		57.0 ^b^	22.3 ^a^	
ANOVA	E	V	E × V	E	V	E × V	E	V	E × V	E	V	E × V
s.e.d	5.10	5.14	7.26	0.88	0.64	0.90	7.52	8.24	11.66	1.92	3.39	4.80
*p*	<0.001	0.001	0.225	<0.001	<0.001	0.019	0.27	<0.001	0.516	<0.001	0.051	0.141
l.s.d (*p* = 0.05)	14.15	11.19	-	2.44	1.39	1.97	-	17.96	-	5.34	7.39	-

Means of the main effects in the same row (or column) that include a common letter are not significantly different at 5%. E, environment; V, variety. The *p* values indicate the statistical significance at 5%; s.e.d, standard error of differences of the means; l.s.d, least significant difference.

**Table 4:** Total soluble solids of capsicum varieties grown under retractable roof production system (RRPS) and open field (OF).

Variety	Total Soluble Solids (°Brix)		
	OF	RRPS	*Mean*
Bloodshot	5.38	6.18	5.78 ^a^
Oxley	6.62	6.57	6.59 ^a^
Quattro	6.38	6.03	6.21 ^a^
Sven	5.38	6.48	5.93 ^a^
*Mean*	5.94 ^a^	6.32 ^a^	
ANOVA	E	V	E × V
s.e.d	0.30	0.38	0.54
*p*	0.273	0.209	0.232
l.s.d (*p* = 0.05)	-	-	-

Means of the main effects in the same row (or column) that include a common letter are not significantly different at 5%. E, environment; V, variety. The *p* values indicate the statistical significance at 5%; s.e.d, standard error of differences of the means; l.s.d, least significant difference.

## Data Availability

Data may be available upon request to the corresponding author.

## Supplementary Materials

The following supporting information can be downloaded at: https://www.mdpi.com/article/10.3390/plants11202794/s1. Table S1. Fertilizing program of the capsicum trial.
