# Supplementary material for: Mitigating the Adverse Effects of Semi-Arid Climate on Capsicum Cultivation by Using the Retractable Roof Production System
Source: Plants (Basel). 2022 Oct 21;11(20):2794. doi: 10.3390/plants11202794 (PMC9608960; doi:10.3390/plants11202794)
Supplement: Supplementary file 1 [file plants-11-02794-s001.zip › plants-1958098-supplementary.pdf]

Table S1. Fertilizing program of the capsicum trial

Fertilising program (kg ha^{-1})			
**Element**	**Basal**	**Fertigation**	**Total**
Nitrogen	30	193	223
Phosphorus	97	44	141
Potassium	166	137	303
Magnesium	30	17	47
Calcium	67	102	169
Copper	0.11	-	0.11
Molybdenum	0.21	-	0.21
Manganese	0.4	-	0.4
Zinc	1.1	-	1.1
